# Supplementary material for: Identification and Empiric Evaluation of New Inhibitors of the Multidrug Transporter P-Glycoprotein (ABCB1)
Source: Int J Mol Sci. 2023 Mar 10;24(6):5298. doi: 10.3390/ijms24065298 (PMC10049699; doi:10.3390/ijms24065298)
Supplement: Supplementary file 1 [file ijms-24-05298-s001.zip › ijms-2139917-supplementary.pdf]

Table S1. Dataset of selective compounds.

SMILE string of compounds	ID	IC$_{50}$ µM)	Reference
O=C(CCc1ccccc1)c1c(OCC(O)C[N+]CCC)cccc1	Q1	1.51	[28]
O=C(CCc1ccccc1)c1c(OCC(O)C[N+](CC)CC)cccc1	Q2	1.7	[28]
O=C(CCc1ccccc1)c1c(OCC(O)C[N+]2CCCCC2)cccc1	Q3	0.8	[28]
O=C(CCc1ccccc1)c1c(OCC(O)C[N+](C(C)C)C(C)C)cccc1	Q4	0.65	[28]
Fc1ccc(N2CC[N+](CC(O)COc3c(C(=O)CCc4ccccc4)cccc3)CC2)cc1	Q5	0.27	[28]
O=C(CCc1ccccc1)c1c(OCC(O)C[N+]2CCCC2)cccc1	Q6	3.6	[28]
O=C(CCc1ccccc1)c1c(OCC(O)CN2CCOCC2)cccc1	Q7	5.83	[28]
O(C(CCc1ccccc1)c1c(OCC(O)C[N+]2CCCCC2)cccc1)C	Q8	0.53	[28]
Fc1ccc(N2CC[N+](CC(O)COc3c(C(OC)CCc4ccccc4)cccc3)CC2)cc1	Q9	0.09	[28]
O(C(CCc1ccccc1)c1c(OCC(O)CN(C(C)C)C(C)C)cccc1)C	Q10	0.65	[28]
Fc1ccc(N2CC[N+](CC(O)COc3c(C(=O)c4ccccc4)cccc3)CC2)cc1	Q11	0.79	[28]
O(CC(O)C[N+]1CCCCC1)c1c(Cc2ccccc2)cccc1	Q12	1.56	[28]
O=C(C)c1c(OCC(O)C[N+]2CCC(O)(c3ccccc3)CC2)cccc1	Q13	1.79	[28]
O=C(CC)c1c(OCC(O)C[N+]2CCC(O)(c3ccccc3)CC2)cccc1	Q14	0.68	[28]
O=C(c1c(OCC(O)C[N+]2CCC(O)(c3ccccc3)CC2)cccc1)c1ccccc1	Q15	0.16	[28]
O(CC(O)CN(C(C)C)C(C)C)c1c(C(O)CCc2ccccc2)cccc1	Q16	3.01	[28]
O=C(Nc1c(N)cc(-c2ccccc2)cc1)c1cc(OC)c(OC)c(OC)c1	Q17	7.2	[26]
Fc1c(NC(=O)c2cc(OC)c(OC)c(OC)c2)ccc(-c2ccccc2)c1	Q18	1.4	[26]
O=C(Nc1c(NC(=O)c2cc(OC)c(OC)c(OC)c2)ccc(-c2ccccc2)c1)C	Q19	1.9	[26]
O=[N+]([O-])c1c(NC(=O)c2cc(OC)c(OC)c(OC)c2)cc(-c2ccccc2)cc1	Q20	0.57	[26]
O=[N+]([O-])c1c(NC(=O)c2cc(OC)c(OC)c(OC)c2)ccc(-c2cc3OCOc3cc2)c1	Q21	0.2	[26]

O=[N+]([O-])c1c(NC(=O)c2cc(OC)c(OC)c(OC)c2)ccc(-c2cc(C(=O)C)ccc2)c1	Q22	0.6	[26]
O=[N+]([O-])c1c(NC(=O)c2cc(OC)c(OC)c(OC)c2)ccc(-c2cc(OC)c(OC)c(OC)c2)c1	Q23	24.3	[26]
O=[N+]([O-])c1cc(-c2ccc(NC(=O)c3cc(OC)c(OC)c(OC)c3)cc2)ccc1	Q24	0.4	[26]
O=[N+]([O-])c1c(NC(=O)c2cc(OC)c(OC)cc2)ccc(-c2ccccc2)c1	Q25	1.3	[26]
O=[N+]([O-])c1c(NC(=O)c2cc(OC)cc(OC)c2)ccc(-c2ccccc2)c1	Q26	6.6	[26]
O=[N+]([O-])c1c(NCc2cc(OC)c(OC)c(OC)c2)ccc(-c2ccccc2)c1	Q27	1.5	[26]
O=[N+]([O-])c1c(C(=O)Nc2cc(OC)c(OC)c(OC)c2)ccc(-c2ccccc2)c1	Q28	2	[26]
O=[N+]([O-])c1c(NC(=O)c2cc(OC)c(O)c(O)c2)ccc(-c2cc(O)c(O)cc2)c1	Q29	>100	[26]
O=[N+]([O-])c1c(NC(=O)c2cc(OC)c(O)c(O)c2)ccc(-c2cc(OC)c(O)c(O)c2)c1	Q30	3.1	[26]
Fc1c(NC(=O)c2cc(OC)c(O)c(O)c2)ccc(-c2ccccc2)c1	Q31	14.1	[26]
O=[N+]([O-])c1c(NC(=O)c2cc(O)c(OC)cc2)ccc(-c2ccccc2)c1	Q32	3.5	[26]
Fc1c(F)ccc(CCN2CCN(c3nc(CN4CCOCC4)nc4n(C)c5c(c(C(=O)N)ccc5)c34)CC2)c1	Q33	>40	[27]
N#Cc1c2ncnc(NCCc3ccccc3)c2n2c1CCCC2	Q34	15.85	[27]
N#Cc1c2ncnc(N(CCc3ccccc3)C)c2n2c1CCCC2	Q35	33.11	[27]
S(=O)(=O)(Nc1ncnc2c(C#N)c3n(c12)CCCC3)Cc1ccccc1	Q36	5.37	[27]
O(C)c1c(OC)cc2c(c1)C[N+](CCN1C(c3ccc(N(C)C)cc3)=Nc3c(OC)cccc3C1=O)CC2	Q37	0.05	[27]
N#Cc1c2ncnc(NCC[N+]3Cc4c(cccc4)CC3)c2n2c1CCCC2	Q38	>50	[27]
O=C(Nc1ccc(-c2nc(N3CC[N+](CCc4ccccc4)CC3)c3n4c(c(C#N)c3n2)CCCC4)cc1)C	Q39	23.44	[27]
Fc1c(F)ccc(CC[N+]2CCN(c3nc(-c4c(C)cccc4)nc4c(C#N)c5n(c34)CCCC5)CC2)c1	Q40	17.78	[27]
O=C1N(CCN2CC[N+](Cc3ccccc3)CC2)C(c2ccc(N(C)C)cc2)=Nc2c1cccc2	Q41	2.1	[27]
O(C)c1c(OC)ccc(CCN(CCN2C(c3ccc(N(C)C)cc3)=Nc3c(C2=O)cccc3)C)c1	Q42	1.9	[27]
O=C1N(CCN2CCN(c3ncccn3)CC2)C(c2ccc(N(C)C)cc2)=Nc2c1cccc2	Q43	0.39	[27]
O(C)c1c(OC)cc2c(c1)C[N+](CCN1C(c3ccc(N(CC)CC)cc3)=Nc3c(C1=O)cccc3)CC2	Q44	1.3	[27]
O(C)c1c(OC)cc2c(c1)C[N+](CCN1C(c3ccc(N(C)C)cc3)=Nc3c(C1=O)ccc(N)c3)CC2	Q45	7.2	[27]
O=C(Nc1ccc(CC[N+]2Cc3c(cc(OC)c(OC)c3)CC2)cc1)/C=C/c1cc(OC)c(OC)c(OC)c1	Q46	0.78	[24]
O=C(Nc1ccc(CC[N+]2Cc3c(cc(OC)c(OC)c3)CC2)cc1)c1cc(OC)c(OC)c(OC)c1	Q47	1.04	[24]
O=C(Nc1ccc(CC[N+]2Cc3c(cc(OC)c(OC)c3)CC2)cc1)c1c2c(cc3c1cccc3)cccc2	Q48	0.66	[24]
O=C(Nc1ccc(CC[N+]2Cc3c(cc(OC)c(OC)c3)CC2)cc1)C(c1ccc(OC)cc1)c1ccc(OC)cc1	Q49	0.3	[24]
O=C(Nc1ccc(CC[N+]2Cc3c(cc(OC)c(OC)c3)CC2)cc1)C1c2c(-c3c1cccc3)cccc2	Q50	0.7	[24]
O=C(Nc1ccc(CC[N+]2Cc3c(cc(OC)c(OC)c3)CC2)cc1)C#Cc1cc(OC)c(OC)c(OC)c1	Q51	0.7	[24]

O=C(Oc1ccc(CC[N+]2Cc3c(cc(OC)c(OC)c3)CC2)cc1)/C=C/c1cc(OC)c(OC)c(OC)c1	Q52	1.4	[24]
O=C(Oc1ccc(CC[N+]2Cc3c(cc(OC)c(OC)c3)CC2)cc1)c1cc(OC)c(OC)c(OC)c1	Q53	0.93	[24]
O=C(Oc1ccc(CC[N+]2Cc3c(cc(OC)c(OC)c3)CC2)cc1)c1c2c(cc3c1cccc3)cccc2	Q54	1.23	[24]
O=C(Oc1ccc(CC[N+]2Cc3c(cc(OC)c(OC)c3)CC2)cc1)C(c1ccc(OC)cc1)c1ccc(OC)cc1	Q55	0.33	[24]
O=C(Oc1ccc(CC[N+]2Cc3c(cc(OC)c(OC)c3)CC2)cc1)C1c2c(-c3c1cccc3)cccc2	Q56	10	[24]
O=C(Oc1ccc(CC[N+]2Cc3c(cc(OC)c(OC)c3)CC2)cc1)C#Cc1cc(OC)c(OC)c(OC)c1	Q57	2	[24]
O(C)c1c(OC)cc(/C=C/CNc2ccc(CC[N+]3Cc4c(cc(OC)c(OC)c4)CC3)cc2)cc1OC	Q58	0.68	[24]
O(C)c1c(OC)cc(CNc2ccc(CC[N+]3Cc4c(cc(OC)c(OC)c4)CC3)cc2)cc1OC	Q59	0.73	[24]
O(C)c1c(OC)cc2c(c1)C[N+](CCc1ccc(NCc3c4c(cc5c3cccc5)cccc4)cc1)CC2	Q60	1.01	[24]
O(C)c1c(OC)cc2c(c1)C[N+](CCc1ccc(NCC(c3ccc(OC)cc3)c3ccc(OC)cc3)cc1)CC2	Q61	0.57	[24]
O(C)c1ccc(C=2Oc3c(c(O)cc(O)c3)C(=O)C=2)cc1	Q62	>25	[48]
Clc1c(C(n2cncc2)(c2ccccc2)c2ccccc2)cccc1	Q63	24.2	[48]
Clc1cc2C(N3CC[N+](C)CC3)=Nc3c(Oc2cc1)cccc3	Q64	>25	[48]
Fc1ccc(C(CCCN2CCC(N3C(=O)Nc4c3cccc4)CC2)c2ccc(F)cc2)cc1	Q65	>25	[48]
O=C(/C=C/c1cc(OC)c(OC)c(OC)c1)N1C(=O)C=CCC1	Q66	>25	[48]
O=C(OC)c1c(NC(=O)c2nc3c(cc2)cccc3)cc(C(=O)Nc2ccc(CC[N+]3Cc4c(cc(OC)c(OC)c4)CC3)cc2)cc1	Q67	>29	[29]
O=C(OC)c1c(NC(=O)c2nc3c(nc2)cccc3)cc(C(=O)Nc2ccc(CC[N+]3Cc4c(cc(OC)c(OC)c4)CC3)cc2)cc1	Q68	>34	[29]
O=C(OC)c1c(NC(=O)c2ncc(C)nc2)cc(C(=O)Nc2ccc(CC[N+]3Cc4c(cc(OC)c(OC)c4)CC3)cc2)cc1	Q69	>57	[29]
O=C(OC)c1c(NC(=O)c2nccnc2)cc(C(=O)Nc2ccc(CC[N+]3Cc4c(cc(OC)c(OC)c4)CC3)cc2)cc1	Q70	>20	[29]
O=C(OC)c1c(NC(=O)c2ncccc2)cc(C(=O)Nc2ccc(CC[N+]3Cc4c(cc(OC)c(OC)c4)CC3)cc2)cc1	Q71	>14	[29]
O=C(Nc1c(OC)ccc(C(=O)Nc2ccc(CC[N+]3Cc4c(cc(OC)c(OC)c4)CC3)cc2)c1)c1cnc2c(c1)cccc2	Q72	>60	[29]
O=C(Nc1ccc(CC[N+]2Cc3c(cc(OC)c(OC)c3)CC2)cc1)c1cc(OC)c(OC)c(NC(=O)c2cnc3c(c2)cccc3)c1	Q73	>17	[29]
O=C(Nc1ccc(CC[N+]2Cc3c(cc(OC)c(OC)c3)CC2)cc1)c1cc(OC)cc(NC(=O)c2cnc3c(c2)cccc3)c1	Q74	>15	[29]
O=C(Nc1c(C)ccc(C(=O)Nc2ccc(CC[N+]3Cc4c(cc(OC)c(OC)c4)CC3)cc2)c1)c1cnc2c(c1)cccc2	Q75	>15	[29]
Fc1cc(F)cc(-c2cc([N+](=O)[O-])c(NC(=O)c3cc(OC)c(OC)c(OC)c3)cc2)c1	Q76	2.6	[26]
Clc1c2C(=O)N(CC[N+]3Cc4c(cc(OC)c(OC)c4)CC3)C(c3ccc(N(C)C)cc3)=Nc2ccc1	Q77	0.16	[27]
O=[N+]([O-])c1cc2C(=O)N(CC[N+]3Cc4c(cc(OC)c(OC)c4)CC3)C(c3ccc(N(C)C)cc3)=Nc2cc1	Q78	0.6	[27]
O(C)c1c(OC)cc2c(c1)C[N+](CCN1C(c3ccc(N(C)C)cc3)=Nc3c(C)cccc3C1=O)CC2	Q79	0.63	[27]
O(C)c1c(OC)cc2c(c1)C[N+](CCN1C(c3cc(CC)ccc3)=Nc3c(C1=O)cccc3)CC2	Q80	0.8	[27]
O(C)c1c(OC)cc2c(c1)C[N+](CCN1C(c3cc4c(cc3)cccc4)=Nc3c(C1=O)cccc3)CC2	Q81	0.85	[27]

O(C)c1c(OC)cc2c(c1)C[N+](CCN1C(c3ccc(C(C)C)cc3)=Nc3c(C1=O)cccc3)CC2	Q82	1	[27]
O(C)c1c(OC)cc2c(c1)C[N+](CCN1C(c3ccc(-c4ccccc4)cc3)=Nc3c(C1=O)cccc3)CC2	Q83	1.03	[27]
Clc1c2N=C(N(CC[N+]3Cc4c(cc(OC)c(OC)c4)CC3)C(=O)c2ccc1)c1ccc(N(C)C)cc1	Q84	1.06	[27]
O(C)c1c(OC)cc2c(c1)C[N+](CCN1C(c3ccc(N(C)C)cc3)=Nc3c(C1=O)cccc3)CC2	Q85	1.07	[27]
O=[N+]([O-])c1cc2N=C(N(CC[N+]3Cc4c(cc(OC)c(OC)c4)CC3)C(=O)c2cc1)c1ccc(N(C)C)cc1	Q86	1.1	[27]
Fc1c(F)ccc(CC[N+]2CCN(c3nc(-c4ccncc4)nc4n5c(c(C#N)c34)CCCC5)CC2)c1	Q87	1.35	[27]
Clc1cc2N=C(N(CC[N+]3Cc4c(cc(OC)c(OC)c4)CC3)C(=O)c2cc1)c1ccc(N(C)C)cc1	Q88	1.35	[27]
O(C)c1c(OC)cc2c(c1)C[N+](CCN1C(c3cc(N(C)C)ccc3)=Nc3c(C1=O)cccc3)CC2	Q89	1.6	[27]
Clc1cc2C(=O)N(CC[N+]3Cc4c(cc(OC)c(OC)c4)CC3)C(c3ccc(N(C)C)cc3)=Nc2cc1	Q90	1.7	[27]
S(=O)(=O)(N1CCCC1)c1cc2c3c(N4CC[N+](CCc5cc(F)c(F)cc5)CC4)ncnc3[nH]c2cc1	Q91	3.32	[27]
Fc1c(F)ccc(CC[N+]2CCN(c3ncnc4n5c(c(C(=O)NCc6ncccc6)c34)CCCC5)CC2)c1	Q92	3.39	[27]
O(C)c1c(OC)cc2c(c1)C[N+](CCN1C(c3cc4OCOc4cc3)=Nc3c(C1=O)cccc3)CC2	Q93	3.7	[27]
O(C)c1c(OC)cc2c(c1)C[N+](CCN1C(c3ccc(N(C)C)cc3)=Nc3c(C1=O)cc(N)cc3)CC2	Q94	2	[27]
O=C(CCc1ccccc1)c1c(OCC(O)CNc2ccccc2)cccc1	Q95	5.5	[28]
O(C)c1c(OC)cc2c(c1)C[N+](CCN1C(c3ccccc3)=Nc3c(C1=O)cccc3)CC2	Q96	8	[27]
O=C(OC)c1ccc(NCC(O)COc2c(C(=O)CCc3ccccc3)cccc2)cc1	Q97	9.61	[28]
Brc1ccc(NC(=O)c2cc(OC)c(OC)c(OC)c2)cc1	Q98	20	[26]
O=C(CC)c1c(OCC(O)C[N+]2CCOCC2)cccc1	Q99	113	[28]
